# Editorial: Drug Hypersensitivity: From Mechanisms to Improved Diagnosis and Standards of Care

**DOI:** 10.3389/fphar.2021.718928

**Published:** 2021-06-17

**Authors:** T. D. Fernandez, M. J. Torres, A. Romano

**Affiliations:** ^1^ Allergy Research Group, Instituto de Investigación Biomédica de Málaga-IBIMA, Málaga, Spain; ^2^ Departamento de Biología Celular, Genética y Fisiología, Universidad de Málaga-IBIMA, Málaga, Spain; ^3^ Allergy Unit, Hospital Regional Universitario de Málaga, Málaga, Spain; ^4^ Departamento de Medicina, Universidad de Málaga-IBIMA, Málaga, Spain; ^5^ Andalusian Centre for Nanomedicine and Biotechnology-BIONAND, Málaga, Spain; ^6^ IRCCS Oasi Maria S.S., Troina, Italy; ^7^ Fondazione Mediterranea G.B. Morgagni, Catania, Italy

**Keywords:** drug, adverse reaction, hypersensitivity, diagnosis, management

Adverse drugs reactions occur in 10–15% of hospitalized patients and cause 3–6% of hospital admissions, constituting adn important public health issue (Doña et al., 2012). Some of them are unpredictable and occur after exposure to a drug at doses normally tolerated. These reactions, called drug hypersensitivity reactions (DHRs), can be immunologically mediated (allergic reactions) or non-immunologically mediated (non-allergic hypersensitivity reactions), which comprise most reactions induced by nonsteroidal anti-inflammatory drugs (NSAIDs) (Pichler, 2019).

Their diagnosis and management is complex due to the lack of knowledge about underlying mechanisms, immunochemistry of the drugs that identify the epitope finally recognizes by the immunological system, variety of clinical symptoms, and heterogeneity among diagnostic protocols used in different centers (Torres et al., 2019). Therefore, an update on drug pharmacology, DHR classifications and mechanisms, and development of new tools and protocols to improve diagnosis and management is essential.

Regarding epidemiology, in a prospective study of 1,553 Kuwaiti patients reporting DHRs, performed by Al-Ahmad et al., NSAIDs and betalactams (BLs) were confirmed as the most commonly implicated drugs. In particular, reactions to BLs were mainly immediate (i.e., occurring within 1 h after drug administration) and the most common symptoms were urticaria, angioedema, and respiratory ones.

In patients with DHRs, diagnostic procedures must be performed by trained personnel in specialized facilities. These procedures include detailed clinical history, *in vivo* tests, mainly skin (STs) and drug provocation tests (DPTs), and *in vitro* assays (Romano et al., 2020). However, the allergy work-up of DHRs inevitably involves costs (Sobrino et al., 2020). In this regard, Sobrino-García et al. analyzed 20 studies regarding the costs of drug hypersensitivity assessment, especially those associated with mislabeling in NSAID or BL hypersensitivity. Their analysis revealed that the diagnosis of DHRs is not expensive, particularly considering the economic and clinical consequences of labeling patients with DHRs. Indeed, proper diagnostic work-ups of DHRs can save money to the health systems.

Nevertheless, the current diagnostic tests are not 100% sensitive (Mayorga et al., 2016). Regarding BLs, the chemical stability of benzylpenicillin reagents used for STs, the best validated *in vivo* method for immediate reactions to BLs, is essential to improve sensitivity. Mayorga et al. noted that butylamine-benzylpenicilloyl conjugates, present in commercial kits for STs, can suffer from an epimerization process, affecting their recognition by patients’ IgE. This phenomenon may have important implications for the reproducibility and sensitivity of both *in vivo* and *in vitro* diagnostic tests.

Traditional diagnostic procedures include many steps, among which the last is DPT, although it is considered the gold standard. In fact, DPT can re-induce the index reaction and, for this reason, it is contraindicated in severe reactions (Romano et al., 2020). Felix and Kuschnir analyzed studies that used a different diagnostic approach, which consisted on performing DPTs with the suspected BLs without previous STs and which demonstrated that this strategy is safe and effective in children with mild non-immediate cutaneous reactions. However, they concluded that further studies involving various populations and age groups are needed to recommend this diagnostic approach, which may still prove to be a feasible and cost-effective strategy in the coming years.

DPT has been recommended not only to identify the culprit drug but also to identify safe alternatives in iodinated contrast media (ICM) allergy (Rosado Ingelmo et al., 2016). Doña et al. prospectively evaluated 101 patients reporting HSRs to ICMs by performing STs with a wide panel of ICMs. In negative STs, a single-blind placebo controlled DPT was carried out. If STs or DPTs were positive, tolerance was assessed with an alternative ICM negative in STs. Among the 101 subjects, 36 (35.6%) were allergic to more than one ICM. The percentage of patients reporting anaphylaxis was higher in patients allergic to multiple ICMs compared with patients allergic to a single ICM (50% vs. 25%).

Regarding *in vitro* tests, the lack of knowledge about the exact epitope, formed by part of the drug and part of the protein to which it is conjugated, able to induce the reaction, can be one of the causes for their non-optimal sensitivity (Ariza et al., 2015). This is specially challenging for clavulanic acid (CLV). Interestingly, Martín-Serrano et al. assessed the suitability of biotinylated analogues of CLV as probes to study protein haptenation by this BL. They demonstrated that these analogues could be valuable to identify protein targets and to get insights into the activation of the immune system by CLV and mechanisms involved in BL allergy. It is also challenging in drugs that are metabolized, like lapatinib, an anticancer drug generally used for breast and lung cancer. Andreu et al. showed that the parent drug and their metabolites have a high affinity to human proteins, especially to human serum albumin (HSA).

All these diagnostic approaches are aimed at identifying the responsible drug, which is essential for a correct patient management. Copaescu et al. provided an updated review of diagnostic methods in delayed T-cell-mediated DHRs, which include *in vivo* and *in vitro* tests. Regarding the latter methods, those most used are lymphocyte transformation test (LTT) and enzyme-linked ImmunoSpot (ELISpot) assay of cytokine release, typically of IFN-γ, after the patient’s peripheral blood mononuclear cells are stimulated with the suspected drug. These tests and HLA-typing have shown to be really useful helping clinicians to prescribe safe and optimal treatments.

Regarding delayed DHRs mainly affecting the skin, it is crucial to consider the importance of a false allergy label due to viral-induced skin lesions leading to the unnecessary avoidance of drugs (Torres et al., 2003). Anci et al. reviewed current knowledge on the different aspects and potential roles of viruses in DHRs. They believed that further studies are needed to understand better the link between viruses and DHRs to improve management of patients presenting skin eruptions during treatments and, above all, to avoid useless drug avoidance, which is related to increased morbidity and mortality.

Once the culprit drug has been identified, the most common treatment is drug avoidance. However, sometimes this is not possible because the drug is the best or the only treatment for a certain pathology. In these situations, desensitization must be the preferred option (Broyles et al., 2020). This process is associated with inhibition of mast cell degranulation and cytokine production. Vultaggio et al. described the involvement of an antigen specific regulation of adaptive response, with an increase in regulatory cytokines, mainly represented by IL-10, and the appearance of IL-35 producing T regulatory cells during desensitization procedure.

Regarding NSAIDs hypersensitivity, the existence of a phenotypic heterogeneity of the NSAID-Exacerbated Respiratory Disease (NERD) has led to establishment of precision medicine strategies tailored/adapted to individual phenotypes/endotypes (Kowalski, 2019). Seong-Dae Woo et al. reviewed the current knowledge on pathophysiologic mechanisms and diagnosis/management approaches of this pathology. Therapeutical options may involve the avoidance of the drug, desensitization to acetylsalicylic acid (ASA), treatment with biologicals, or even dietary interventions.

There is still a lack of information on the complete molecular picture of the pathogenic mechanisms of NERD, although the involvement of platelet-adherent leukocytes and integrins has been described (Laidlaw et al., 2012). Jurado-Escobar et al. investigated such involvement in NSAID-induced urticaria/angioedema (NIUA), the most frequent clinical phenotype. Their results supported the participation of platelet-adherent leukocytes and integrins in this pathology and provided a link between these cells and arachidonic acid metabolism, although, unlike NERD, in NIUA they did not find a systemic imbalance in the frequency of CD61^+^ cells/integrin expression or levels of LTE4.

Finally, Ghada et al. reviewed the current knowledge on hypersensitivity to rituximab, a chimeric monoclonal antibody used to treat various lymphoid malignancies, lymphoproliferative diseases, and rheumatologic disorders. Increased use of rituximab has been associated with an increase in several types of hypersensitivity reactions. The authors made a great effort to review several aspects of hypersensitivity reactions to this drug, in particular the clinical presentations, pathogenic mechanisms, and management of the reactions, including rapid desensitization.

In summary, in this Research Topic it has been revised the current knowledge on all aspects related to DHRs, especially epidemiology, pathogenic mechanisms, immunochemistry of the drugs, diagnosis, and management, including the latest developments.
